# Repercussions of Denosumab in Patients With Giant Cell Tumor of Bone: An Observational Study

**DOI:** 10.7759/cureus.48702

**Published:** 2023-11-12

**Authors:** Binay K Rath, Amrit G, Pranati Mohanty, Aakankshya Tripathy, Jyoti Prakash Sahoo

**Affiliations:** 1 Orthopaedics, SCB Medical College and Hospital, Cuttack, IND; 2 Pathology and Laboratory Medicine, SCB Medical College and Hospital, Cuttack, IND; 3 Pharmacology, SCB Medical College and Hospital, Cuttack, IND; 4 Pharmacology, Kalinga Institute of Medical Sciences, Bhubaneswar, IND

**Keywords:** neoadjuvant therapies, pain on vas, stromal cells, giant cells, bone tumors, musculoskeletal tumor society rating scale, visual analog scale, rank and rankl, denosumab and giant cell tumor, giant cell tumour of bone

## Abstract

Background and objectives: The advent of denosumab has rendered giant cell tumors (GCT) of the bone amenable. It makes sense to evaluate its effect on the patient's symptoms and the histopathological outcomes. The aim of the study is to ascertain the effect of 24-week neoadjuvant denosumab therapy on the following parameters: visual analogue scale (VAS), musculoskeletal tumor society (MSTS) scores, tumor size, and the number of giant and stromal cells.

Material and methods: This observational study was conducted from February 2022 to April 2023 at SCB Medical College and Hospital, India. Fifty-four GCT participants had their VAS and MSTS scores assessed at baseline and then every four weeks for the next 24 weeks. At 24 weeks, changes in their tumor size and the number of giant and stromal cells per high-power field (hpf) were also evaluated.

Results: Fifty-four (82%) out of the 66 enrolled participants completed the study. Among those 54, 29 (54%) participants were female. The study population had a mean age of 39.0 ± 4.7 years. The median VAS scores were (female: 7.0, male: 7.0; p = 0.51) at baseline and (female: 2.0, male: 2.0; p = 0.39) at 24 weeks. The median MSTS scores at baseline and 24 weeks were (female: 8.0, male: 8.0; p = 0.41) and (female: 15.0, male: 16.0; p = 0.66), respectively. The median reductions in tumor size, the number of giant, and stromal cells (per hpf) were (female: 6.0 mm, male: 5.0 mm; p = 0.11), (female: 25, male: 27; p = 0.07), and (female: 1200, male: 2100; p < 0.001), respectively.

Conclusion: After receiving neoadjuvant denosumab for 24 weeks, the study participants' clinical symptoms and histological indicators improved. With the exception of the stromal cells, there was no statistically significant difference between the male and female participants.

## Introduction

Giant cell tumor (GCT), a common benign bone tumor, is responsible for 4-7% of primary bone tumors [1,2]. Usually, the distal femur, proximal tibia, distal radius, and proximal humerus are affected at their metaphyseal areas [3]. It commonly manifests in the third and fourth decades of life, with a slightly higher female preponderance [1,4]. The surgical techniques, such as en-bloc resection, intralesional curettage, extended curettage with bone grafting, and arthrodesis, tackle the bulk of GCT cases [1,3-4]. With those methods, the functionality of the adjacent joint stays unaltered, but the recurrence rate of GCT increases. Although wide-margin excision reduces the likelihood of tumor recurrence, it worsens functional outcomes [3,5,6]. Denosumab, a completely human monoclonal antibody, targets the receptor activator of the nuclear factor kappa-B ligand (RANKL). Bone resorption is exacerbated by the RANKL's contribution to osteoclast formation [5,7].

Denosumab administered prior to surgery lessens the tumor size and likelihood of its recurrence [8,9]. Conversely, the study by Li et al. [10] claimed that neoadjuvant denosumab therapy raises the risk of GCT recurrence and malignancy. The effects of preoperative denosumab on symptoms and histological outcomes differ according to the patient's age, gender, and bone mineral density [11-13]. Studies comparing denosumab's effects on males and females with GCT are scarce in the Indian subcontinent. We mapped this study to evaluate the effect of 24-week neoadjuvant denosumab on pain and overall function in GCT patients with the visual analog scale (VAS) and the musculoskeletal tumor society (MSTS) scale [14]. We also measured the changes in tumor size and the number of giant and stromal cells after 24 weeks.

## Materials and methods

This observational study was carried out from February 2022 to April 2023 at SCB Medical College, Cuttack, India. Prior to study commencement, we received ethical approval (IEC application no. 1030 dated February 11, 2022) from the SCB Medical College's Institutional Ethics Committee. Prior to being enrolled, all participants gave their consent. Adult patients of both sexes, aged 18-50 years, with a body weight > 45 kg and a body mass index (BMI) > 18.5 kg/m2 at baseline and a clinical diagnosis of GCT of bone (confirmed by radiography and histopathology), were included in the study. Individuals with pathological fractures, osteoporosis, hypocalcemia, osteonecrosis of the jaw, osteosarcoma, any secondary malignancy within the past five years, GCT at the spine or sacrum (difficulty in surgical access), multifocal or recurrent GCT, pregnant or lactating women, and those unwilling to give consent were excluded from the study.

The primary objectives were to evaluate changes in the female and male participants' VAS and MSTS scores after 24 weeks of denosumab therapy. The proportion of male and female participants with GCT at different sites, as well as changes in the tumor size and the number of giant and stromal cells per high-power field (hpf) after 24 weeks, were the secondary objectives.

We sorted the enrolled participants by gender. This study used the convenience sampling approach. Each of them underwent systemic, musculoskeletal, hematological, and radiological assessments. Following the Campanacci [15] classification of the tumors, we performed a core needle biopsy close to the anticipated line of incision. In order to validate the diagnosis, calculate the tumor size, and determine the number of giant and stromal cells per unit hpf, the sample was sent for histological examination. Each participant received subcutaneous injections of denosumab 120 mg on the abdomen or anterior side of the thigh every four weeks for 24 weeks. They also got 500 mg of elemental calcium tablets and 400 IU of vitamin D capsules once daily.

At baseline (visit 1), plus at every follow-up appointment (visits 2-7) at four, eight, 12, 16, 20, and 24 weeks after the baseline visit, we evaluated the VAS and MSTS scores. The MSTS [14] scale is used to evaluate upper or lower limb performance in GCT patients. This scale encompasses six domains: pain, functional activity, positioning, dexterity, muscle power, and emotional acceptance. For each domain, the scores range from 0 to 5, reflecting the worst to best responses. The total added score spans from 0 to 30. Higher scores indicate improved performance. The VAS is one of the most frequently used tools to measure pain. This emoji-based response scale ranges from 0 to 10, expressing the best to worst pain perception [16].

We assessed the normality of the data distribution with the Shapiro-Wilk test. The continuous data were portrayed as median (interquartile range) and the categorical data as frequency (%). The continuous and categorical data were analyzed using the Chi-square and Wilcoxon tests. For data analysis, we used the R software (version 4.2.1, R Foundation for Statistical Computing, Vienna, Austria) [17].

## Results

We scrutinized 103 GCT patients for this study. Twenty-seven individuals had recurrent GCT; one patient had GCT in three different bones (the right humerus, left femur, and tibia); three were post-menopausal women receiving injections of teriparatide for osteoporosis; and six patients declined to provide consent. The remaining 66 were enrolled. Twelve patients did not come for all the follow-up appointments. Once the remaining 54 participants finished the study, their data were analyzed. Table 1 displays the study population's baseline demographics and clinical parameters. The study participants had a median age of 39.0 (33.3-42.0) years. The median baseline VAS and MSTS scores were 7.0 (6.0-7.1) and 8.0 (7.0-9.0), respectively.

**Table 1:** Baseline demographic and clinical parameters of the study population The categorical and continuous data were expressed as n (%) and median (interquartile range), respectively. BMI: body mass index; VAS: visual analog scale for pain (10-point scale); MSTS: musculoskeletal tumor society scale (range of scores: 0 to 30); and hpf: high-power field.

Parameters	Total (n = 54)	Female (n = 29)	Male (n = 25)	p-value
Age (years)	39.0 (33.3–42.0)	39.0 (37.0–42.0)	38.0 (32.0–41.0)	0.64
Weight (kg)	55.8 (47.2–68.4)	51.3 (47.2–60.5)	59.9 (48.7–68.4)	0.33
BMI (kg/m^2^)	24.4 (22.1–27.8)	24.1 (22.3–26.6)	24.6 (22.9–27.8)	0.68
VAS score	7.0 (6.0–7.8)	7.0 (7.0–8.0)	7.0 (6.0–7.0)	0.51
MSTS score	8.0 (7.0–9.0)	8.0 (8.0–9.0)	8.0 (7.0–9.0)	0.41
Campanacci grading				
Grade II	24 (44.4%)	13 (44.8%)	11 (44.0%)	0.46
Grade III	30 (55.6%)	16 (55.2%)	14 (56.0%)	
Tumor size (mm)	66.5 (42.0–83.5)	66.0 (43.0–82.0)	68.0 (42.0–90.0)	0.69
Giant cell (per hpf)	30.0 (22.3–46.5)	30.0 (23.0–47.0)	30.0 (22.0–45.0)	0.90
Stromal cell (per hpf)	3200 (1850–3950)	2800 (1200–3800)	3500 (3000–4000)	0.04

**Figure 1:**
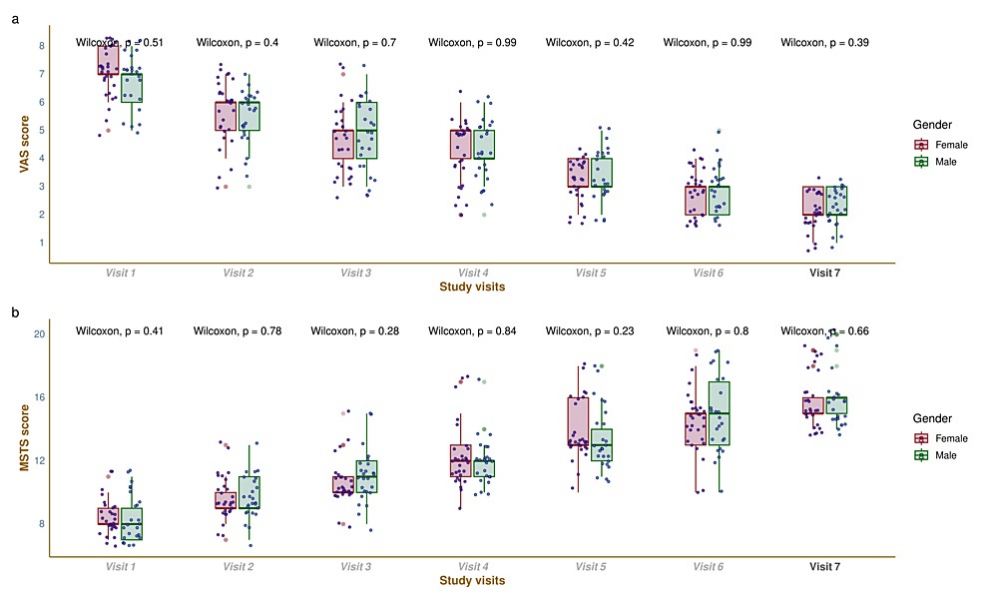
The VAS and MSTS scores of the study participants The box-whisker plots, along with jitter plots, illustrate the VAS and MSTS scores of the study participants throughout the study duration. The study visits 1-7 refer to the visits at the baseline, four, eight, 12, 16, 20, and 24 weeks, respectively. The Wilcoxon test was harnessed to assess the intergroup differences at all time points. Part A depicts the visual analog scale (VAS) scores for pain of the study participants. The higher scores imply more severe pain. Part B shows the Musculoskeletal Tumor Society scoring system (MSTS) scores of the study participants. The higher the score, the more improved the overall function.

The VAS and MSTS scores throughout the study duration are shown in Figure 1. The VAS scores for females and males were 7.0 (7.0-8.0) and 7.0 (6.0-7.0) at baseline (p=0.51), 6.0 (5.0-6.0) and 6.0 (5.0-6.0) at week four (p=0.40), 5.0 (4.0-5.0) and 5.0 (4.0-6.0) at week eight (p=0.70), 5.0 (4.0-5.0) and 4.0 (4.0-5.0) at week 12 (p=0.99), 3.0 (3.0-4.0) and 3.0 (3.0-4.0) at week 16 (p=0.42), 3.0 (2.0-3.0) and 3.0 (2.0-3.0) at week 20 (p=0.99), and 2.0 (2.0-3.0) and 2.0 (2.0-3.0) at week 24 (p=0.39), respectively (Figure 1a). The changes in VAS scores among female and male participants were -5.0 (-5.0 to -4.0) and -4.0 (-5.0 to -4.0), respectively (p=0.08). The pain scores assessed with VAS were reduced in both groups. None of the intergroup differences were statistically significant at any time points of assessment. The MSTS scores for females and males were 8.0 (8.0-9.0) and 8.0 (7.0-9.0) at baseline (p=0.41), 9.0 (9.0-10.0) and 9.0 (9.0-11.0) at week four (p=0.78), 10.0 (10.0-11.0) and 11.0 (10.0-12.0) at week eight (p=0.28), 12.0 (11.0-13.0) and 12.0 (11.0-12.0) at week 12 (p=0.84), 13.0 (13.0-16.0) and 13.0 (12.0-14.0) at week 16 (p=0.23), 15.0 (13.0-15.0) and 15.0 (13.0-17.0) at week 20 (p=0.80), and 15.0 (15.0-16.0) and 16.0 (15.0-16.0) at week 24 (p=0.66), respectively (Figure 1b). The changes in MSTS scores at week 24 among female and male participants were 7.0 (6.0-8.0) and 7.0 (6.0-9.0), respectively (p=0.06). The overall functions assessed with the MSTS scale improved in both groups. Nonetheless, the intergroup differences were not statistically significant at any time points of assessment.

**Figure 2:**
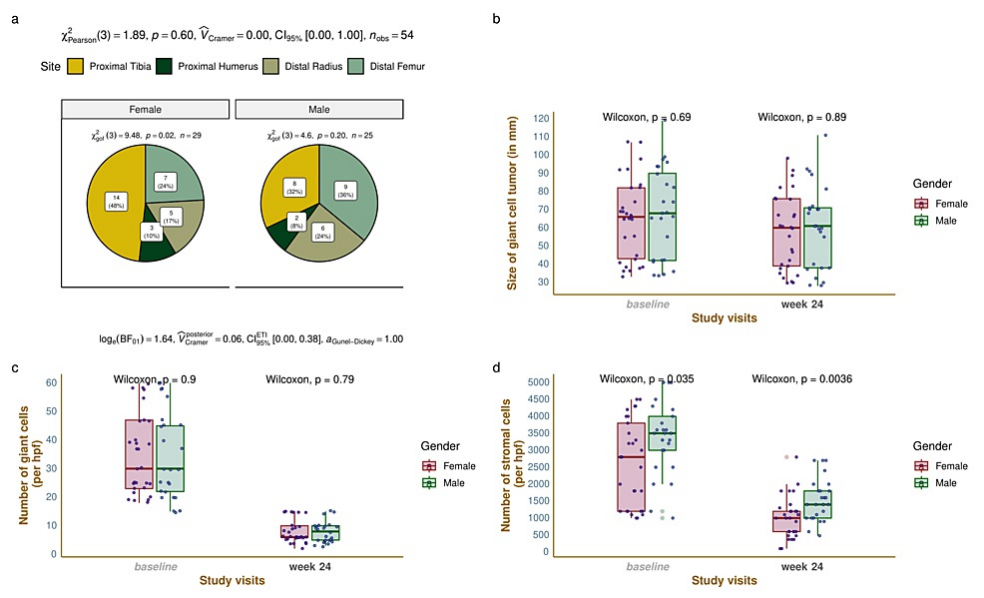
The histopathological findings of GCT This figure shows the sites of giant cell tumors (GCT) of bone and the histopathological findings of the study participants. The pie diagram in part A depicts the number and proportions of different sites of GCT in females and males. The box-whisker plus jitter plots in parts B, C, and D illustrate the size of GCT and the number of giant and stromal cells in female and male participants at baseline and 24 weeks.

Figure 2 displays the sites and the size of GCT, the number of giant and stromal cells at baseline, and the final follow-up visit at week 24. The pie diagrams in Figure 2a demonstrate that the most common site for GCT was the proximal tibia (22; 41%), followed by the distal femur (16; 30%), the distal radius (11; 20%), and the proximal humerus (5; 9%). The order of the GCT sites was the same for both females (p=0.02) and males (p=0.20). However, intragroup comparison yielded a nonsignificant difference (p=0.60). The box-whisker plots in Figure 2b depict that the median size of GCT (in mm) among female and male participants was 66.0 (43.0-82.0) and 68.0 (42.0-90.0) at baseline (p=0.69), and 60.0 (39.0-76.0) and 61.0 (38.0-71.0) at week 24 (p=0.89), respectively. The changes in tumor size after 24 weeks of denosumab therapy were -6.0 (-8.0 to -4.0) and -5.0 (-8.0 to -4.0), respectively (p=0.11). The box-whisker plots in Figure 2c show that the median number of giant cells (per hpf) among female and male participants were 30.0 (23.0-47.0) and 30.0 (22.0-45.0) at baseline (p=0.90), and 6.0 (6.0-10.0) and 8.0 (5.0-10.0) at week 24 (p=0.79), respectively. The median changes in giant cell numbers (per hpf) after 24 weeks of denosumab were -25.0 (-33.0 to -19.0) and -27.0 (-35.0 to -16.0), respectively (p=0.07). The box-whisker plots in Figure 2d show that the median number of stromal cells (per hpf) among female and male participants were 2800 (1200-3800) and 3500 (3000-4000) at baseline (p=0.035), and 1000 (600-1200) and 1400 (1000-1800) at week 24 (p=0.004), respectively. The median changes in stromal cell numbers (per hpf) after 24 weeks were -1200 (-2500 to -830) and -2100 (-2400 to -1200), respectively (p<0.001).

## Discussion

This observational study investigated the effect of 24-week neoadjuvant denosumab on pain, overall function, tumor size, and histopathology (i.e., the number of giant and stromal cells) among female and male GCT patients. Every four weeks, denosumab 120 mg was injected subcutaneously into each subject. They also got 500 mg of elemental calcium tablets and 400 IU of vitamin D capsules once daily. We assessed the VAS and MSTS scores at each visit. We also computed the proportions of male and female patients with GCT at different sites, changes in tumor size, and the number of giant and stromal cells per unit hpf. Both females and males exhibited lower pain levels and better overall functions. Proximal tibia accounted for 41% of GCT cases. Following a 24-week course of denosumab, the GCT size and the count of giant and stromal cells dropped.

After 24 weeks of denosumab therapy, the severity and duration of pain sensations improved. According to the study by Traub et al. [18], neoadjuvant denosumab lowers pain in GCT patients. Neoadjuvant denosumab therapy for GCT ameliorates patients' overall function as measured by MSTS [14] scores, according to research by Traub et al. [18] and a systematic review by Luengo-Alonso et al. [19]. Our findings match these studies. In our study population, the proximal tibia (22; 41%), distal femur (16; 30%), distal radius (11; 20%), and proximal humerus (5; 9%) were the most frequent sites for GCT. After 24 weeks, the tumor size and the number of stromal giant cells plummeted. According to the study by Engellau et al. [20], preoperative denosumab is advantageous for surgically excising the tumor mass since it reduces the size of the GCT. According to a study by Liu et al. [21], the tibia and femur are the most common sites for GCT. It has been suggested in recent studies by Hayashida et al. [13] and Lejoly et al. [22] that neoadjuvant denosumab mitigates the numbers of giant and stromal cells in GCT patients. The findings of our study matched those of these studies.

This study evaluated the clinical symptoms and histopathological parameters of patients with GCT. This study has a few limitations. Probably due to the coronavirus disease 2019 (COVID-19) pandemic, the sample size was relatively modest. The sample size for our study was further reduced by excluding individuals who had multifocal or recurrent GCT. The effects of calcium and vitamin D supplementation on the study's goals were not addressed. We did not follow up with the study participants following their surgeries. We are currently undertaking a separate study using neoadjuvant denosumab to analyze patient’s quality-of-life and GCT recurrence rates.

## Conclusions

We conclude that improvements in pain symptoms, overall function, tumor size, and histological characteristics occurred after a 24-week course of neoadjuvant denosumab. Neoadjuvant denosumab was found to be advantageous in several aspects instead of simply shrinking the tumor size for surgical resection. However, there was no statistically significant difference between the females and males, except for the stromal cells. We warrant more such studies with a larger sample size and a longer study duration.
